# Particle Size Distribution in Aluminum Manufacturing Facilities

**DOI:** 10.5539/ep.v3n4p79

**Published:** 2014-09-24

**Authors:** Sa Liu, Elizabeth M. Noth, Christine Dixon-Ernst, Ellen A. Eisen, Mark R. Cullen, S. Katharine Hammond

**Affiliations:** 1 Division of Environmental Health Sciences, School of Public Health, University of California, Berkeley, CA, USA; 2 Alcoa, Pittsburgh, PA, USA; 3 Department of Internal Medicine, Stanford University, Stanford, CA, USA

**Keywords:** particle size distribution, aluminum, smelter, fabrication

## Abstract

As part of exposure assessment for an ongoing epidemiologic study of heart disease and fine particle exposures in aluminum industry, area particle samples were collected in production facilities to assess instrument reliability and particle size distribution at different process areas. Personal modular impactors (PMI) and Minimicro-orifice uniform deposition impactors (MiniMOUDI) were used. The coefficient of variation (CV) of co-located samples was used to evaluate the reproducibility of the samplers. PM_2.5_ measured by PMI was compared to PM_2.5_ calculated from MiniMOUDI data. Mass median aerodynamic diameter (MMAD) and concentrations of sub-micrometer (PM_1.0_) and quasi-ultrafine (PM_0.56)_ particles were evaluated to characterize particle size distribution. Most of CVs were less than 30%. The slope of the linear regression of PMI_PM_2.5_ versus MiniMOUDI_PM_2.5_ was 1.03 mg/m^3^ per mg/m^3^ (± 0.05), with correlation coefficient of 0.97 (± 0.01). Particle size distribution varied substantively in smelters, whereas it was less variable in fabrication units with significantly smaller MMADs (arithmetic mean of MMADs: 2.59 μm in smelters vs. 1.31 μm in fabrication units, p = 0.001). Although the total particle concentration was more than two times higher in the smelters than in the fabrication units, the fraction of PM_10_ which was PM_1.0_ or PM_0.56_ was significantly lower in the smelters than in the fabrication units (p < 0.001). Consequently, the concentrations of sub-micrometer and quasi-ultrafine particles were similar in these two types of facilities. It would appear, studies evaluating ultrafine particle exposure in aluminum industry should focus on not only the smelters, but also the fabrication facilities.

## 1. Introduction

Increased risk of cardiovascular disease (CVD) related to particulate matter (PM) in air pollution has become a major public health concern in the US and worldwide. Exposures have been associated with sub-clinical markers of vascular function (Fang et al., 2008; Simkhovich et al., 2008; Fang et al., 2009), hospital admissions (Dominici et al., 2006; Peng et al., 2008) and mortality in both men and women (Dockery et al. 1993, Miller et al. 2007, Ostro et al. 2007). Most attention has focused on fine particles (PM_2.5_), with their ability to penetrate into the alveolar region of the lungs (Laden et al., 2000; Dominici et al., 2006; Ostro et al., 2008; Ostro et al., 2009; Pope et al., 2009). However, evidence regarding occupationally-related heart disease risk associated with PM exposure is scant. One reason for this is historically inadequate characterization of workplace particulate matter such that fine particles (PM_2.5_) have not been regulated separately, hence rarely sampled. As part of an ongoing epidemiologic study evaluating the association between CVD and exposure to PM_2.5_ in a cohort of approximately 12,000 aluminum workers, we collected extensive personal and area samples in the aluminum production facilities in 2009 – 2010 using both traditional closed-face cassettes (for total particles) and size-specific cascade impactors (for PM_2.5_). Personal samples were used to estimate individual workers exposure to PM_2.5_ and results were published recently (Noth et al. 2014). Area samples were collected to test instrument performance in the field setting, cross-check the performance of the cascade impactors and explore particle size distributions at different production areas.

This manuscript reported the results of area samples. Two types of cascade impactors were used, Personal Modular Impactor (PMI) (SKC, Eighty Four, PA), which collects particles in three size factions: <2.5 μm, 2.5–10 μm, and >10 μm, and a six-stage minimicro-orifice uniform deposition impactor (MiniMOUDI)(MSP Corporation, Shoreview, MN) with aerodynamic cut-offs of 0.56 μm, 1.0 μm, 1.8 μm, 3.2 μm, 5.6 μm, 10 μm and. The MiniMOUDI provides more detailed information on particle size distribution comparing to the PMI and is able to measure particles in sub-micrometer ranges. However the cost of the MiniMOUDI, along with other factors such as bulkiness and feasibility of being used as a personal sampling device in a work environment, limited its use in personal sample collection in our study. Instead, PMIs were used to collect personal samples side-by-side with cassettes to understand the relationship between PM_2.5_ and total particles that have been traditionally measured by cassette. In this manuscript we examined the reproducibility of co-located impactor samplers and compared PMI measurements with co-located MiniMOUDI samples to ensure that the PMI provided valid PM_2.5_ measurements. We also analyzed particle size distributions at different production areas based on MiniMOUDI measurements.

## 2. Methods

### 2.1 Sample Collection

Samples were collected at eight aluminum facilities in 2009 – 2010. These facilities were selected to encompass different manufacturing processes including refining, smelting and fabricating. Sampling locations were determined by a senior industrial hygienist from the company to cover the jobs with the most number of workers or with potential high particle exposures. Because this was an exposure assessment study tailored for the epidemiologic study, the number of replicates at each location and number of locations to be monitored were carefully balanced to serve both the needs of to test instruments and to capture the variations within and between locations. Area samples were collected in triplicate (PMI, MiniMOUDI and cassette) with a sampling board behind the samplers to reduce the influence of current. Duplicate and triplicate samples were collected at a subset of locations to evaluate the precision of the instruments. Samples were collected in each facility under the direction of certified industrial hygienists. Flow rates were 2.0 and 3.0 liters per minute for MiniMOUDI and PMI, respectively, and were checked at the beginning and end of each sampling period. Samples were analyzed gravimetrically according to the National Institute for Occupational Safety and Health (NIOSH) analytical method 0500 (NIOSH 1994) at an American Industrial Hygiene Association accredited industrial hygiene laboratory. Results reported as less than 0.01 mg/m^3^ for each individual impactor stage (the limit of detection, LD) was assigned a value of 0.007 (0.01/√2). Some PMI samplers were oiled for the first stage (PM > 10 μm stage) to reduce particle bouncing and thus had no results for PM > 10 μm.

### 2.2 Data Analysis

Coefficient of variation (CV) of co-located samples was used to evaluate the reproducibility of impactor samplers (relative difference was used for duplicate samples). For MiniMOUDI samplers, we used PM_10_ and PM_3.2_ (MM_PM_10_ and MM_PM_3.2_) as metrics. For PMI samplers, the reproducibility was evaluated by PM_10_ and PM_2.5_ (PMI_PM_10_ and PMI_PM_2.5_). These values are directly measured gravimetrically from individual stages of MiniMOUDI and PMI samplers. PM_3.2_ was chosen as a metric for the MiniMOUDI because it was the stage closest to PM_2.5_. In order to evaluate the validity of the PMI sampler in measuring PM2.5, we treated the MiniMOUDI as the gold standard (as it measures particles in seven size ranges and gives much detailed particle size distribution information) and compared measured PMI_PM_2.5_ to PM_2.5_ estimated from MiniMOUDI data (MM_PM_2.5_). Mass from each MiniMOUDI stage was used to calculate mass fraction of the total mass. The cumulative mass fractions were plotted on a log-probability graph against mid-point of each particle size interval to obtain the particle size distribution; MM_PM_2.5_ was calculated from the distribution. Furthermore, the particle distribution on the log-probability plot was used to determine mass median aerodynamic diameter (MMAD, μm) and geometric standard deviation (GSD) of the size distribution. MMAD is the particle aerodynamic diameter corresponding to 50% of cumulative mass, while GSD is determined by the ratio of particle sizes associated with 50% to 16% of cumulative mass. We used MMAD and GSD to characterize particle size distributions measured by MiniMOUDI across production areas. Concentrations of PM_1.0_ (sub-micrometer) and PM_0.56_ (quasi-ultrafine) particles as measured by MiniMOUDI were also used to compare particle size characteristics across locations.

## 3. Results

A total of 80 PMI and 62 MiniMOUDI samples were collected at 44 production areas/subareas. MiniMOUDI and PMI samplers were co-located at 32 locations. Less than 0.5% of MiniMOUDI data were below detection limit (particle mass concentration < 0.01 mg/m^3^ on a stage), whereas about 14% of PMI data were less than detectable. Particle sizes were approximately lognormally distributed.

### 3.1 Reproducibility of Co-Located Impactors

**Figure 1:**
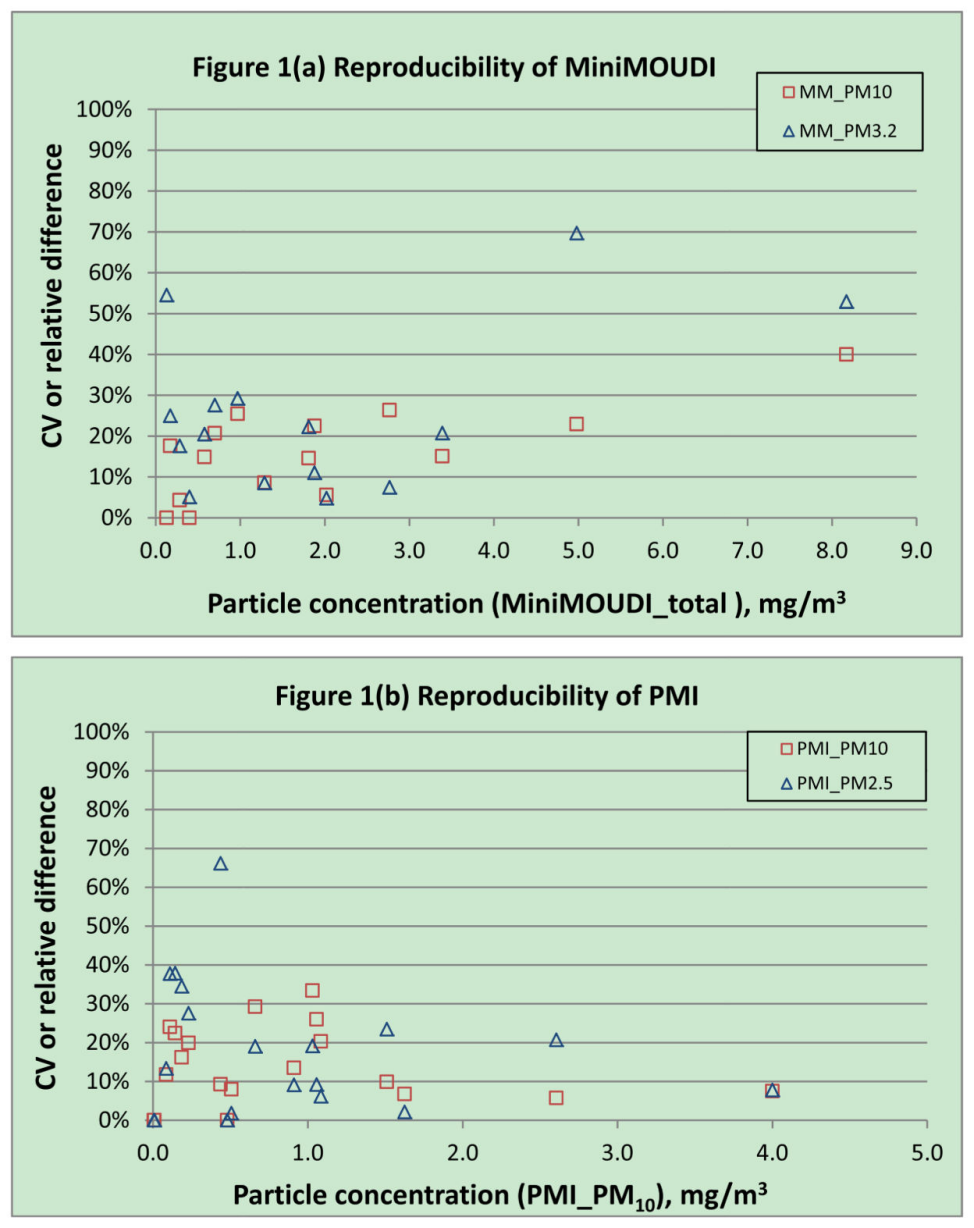
Reproducibility of impactors. (a) MiniMOUDI samples, ordered by MiniMOUDI_total; (b) PMI samples, ordered by PMI_PM_10_ as some PMI samples were oiled for the first stage to reduce particle bouncing and thus did not have PMI_total.

Multiple MiniMOUDI samples were collected at 15 locations, with seven duplicates and eight triplicates. Reproducibility was found to be moderate to high for this type of impactor. Precision for MM_PM_10_ appeared to be better than that for MM_PM_3.2_, with only one relative difference for MM_PM_10_ above 30%, whereas three relative differences for MM_PM_3.2_ were above 30% (Figure 1(a)). Figure 1(a) also illustrates that all relative differences exceeding 30% were from samples with either lowest (one sample) or highest (two samples) observed particle concentrations. Twenty sets of concurrent PMI samples were collected, with seven duplicates and thirteen triplicates. PMI samples appeared to be as precise as co-located MiniMOUDI samples (Figure 1(b)), with one CV for PMI_PM_10_ and four CVs (or relative differences) for PMI_PM_2.5_ above 30%. High CVs for PMI_PM_2.5_ were all from the samples with low particle concentrations.

### 3.2 Comparison of PM_2.5_ between PMI and MiniMOUDI

**Figure 2:**
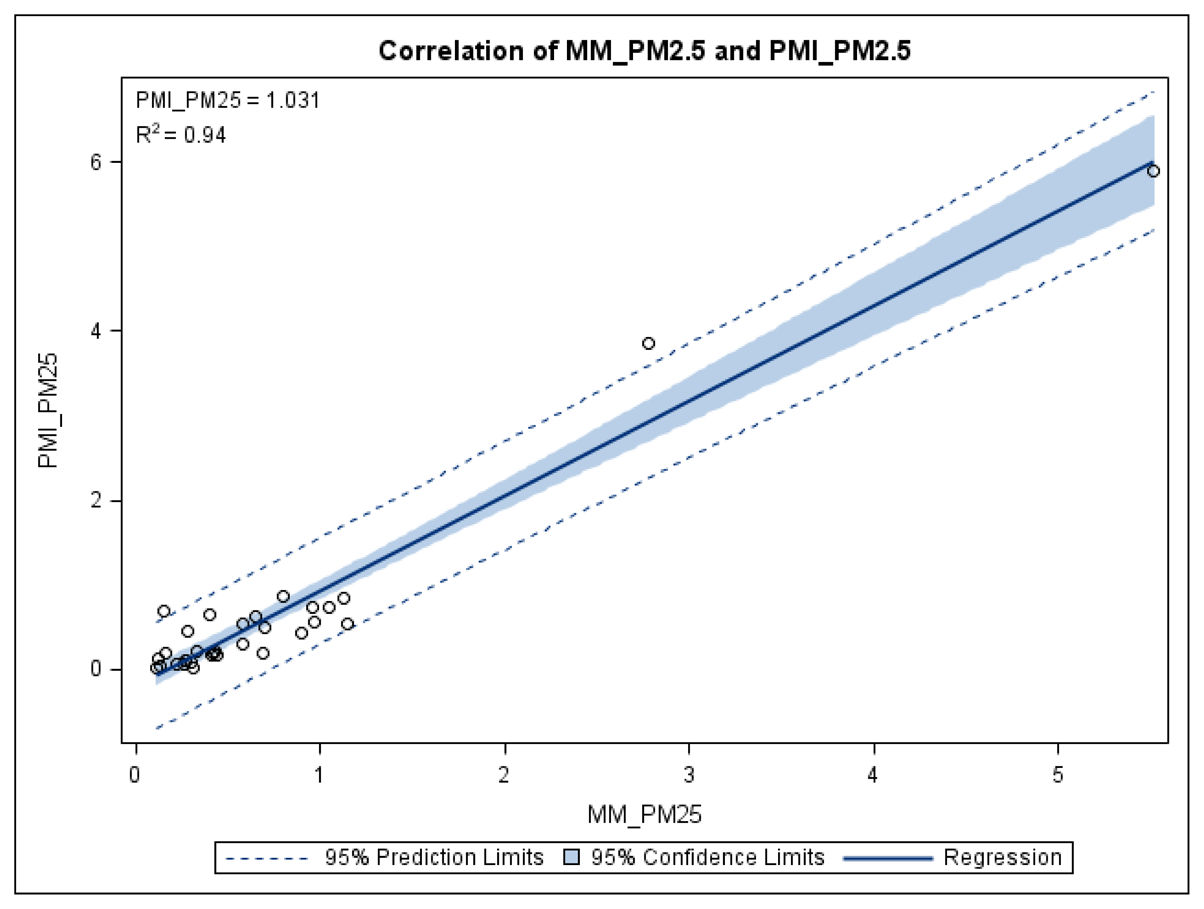
Correlation between PM_2.5_ measured by PMI and calculated from MiniMOUDI data. Each data point is an average of co-located samples from same type of samplers. The equations on the upper left corner are the slope and the R^2^ of the regression line.

PMI and MiniMOUDI samples were collected side-by-side at 32 locations. Figure 2 illustrates the degree of correlation among PMI_PM_2.5_ (measured) and MM_PM_2.5_ (calculated). Measured PMI_PM_2.5_ from individual samples ranged from less than 0.01 mg/m^3^ to 5.9 mg/m^3^. The slope of the regression line is 1.03 (± 0.05), and the Pearson correlation coefficient (r) is 0.97 (± 0.01); this correlation coefficient drops to 0.71 if two data points with high PM_2.5_ concentration were omitted.

### 3.3 Particle Size Distribution at Different Production Areas

We measured particle size distribution by MiniMOUDI at 31 production areas/subareas. The calculated individual MMAD ranged from 0.39 to 6.66 μm. MMADs from co-located samples were averaged to characterize particle size distribution at each location and reported in Table 1. Our data indicate substantial variations in particle concentration and size distribution across production areas, especially those in smelters. MiniMOUDI_total in smelters ranged from 0.28 mg/m^3^ to 4.98 mg/m^3^, with an arithmetic mean (AM) of 1.50 mg/m^3^ (Table 1). MMAD varied considerably in smelters (range: 0.68 – 5.57 μm, AM: 2.59 μm). Samples collected in the bath crushing area, where raw and recycled material was crushed prior to being mixed with other raw materials (alumina, cryolite) and added to the smelting pots, were characterized by both large particle size and high particle air concentration. In Green Anode where the anode-making process starts, raw material such as calcined coke and binders are blended in heated mixing boxes, and thus large particles and high particle air concentration were observed. However, smaller sized particles were measured in the Baked Anode area, with MMAD less than 1.05 μm. Particle size and air concentration were similar in potrooms in the two smelters (Facility B and C), suggesting these characteristics may be comparable in potrooms across facilities.

**Figure 3:**
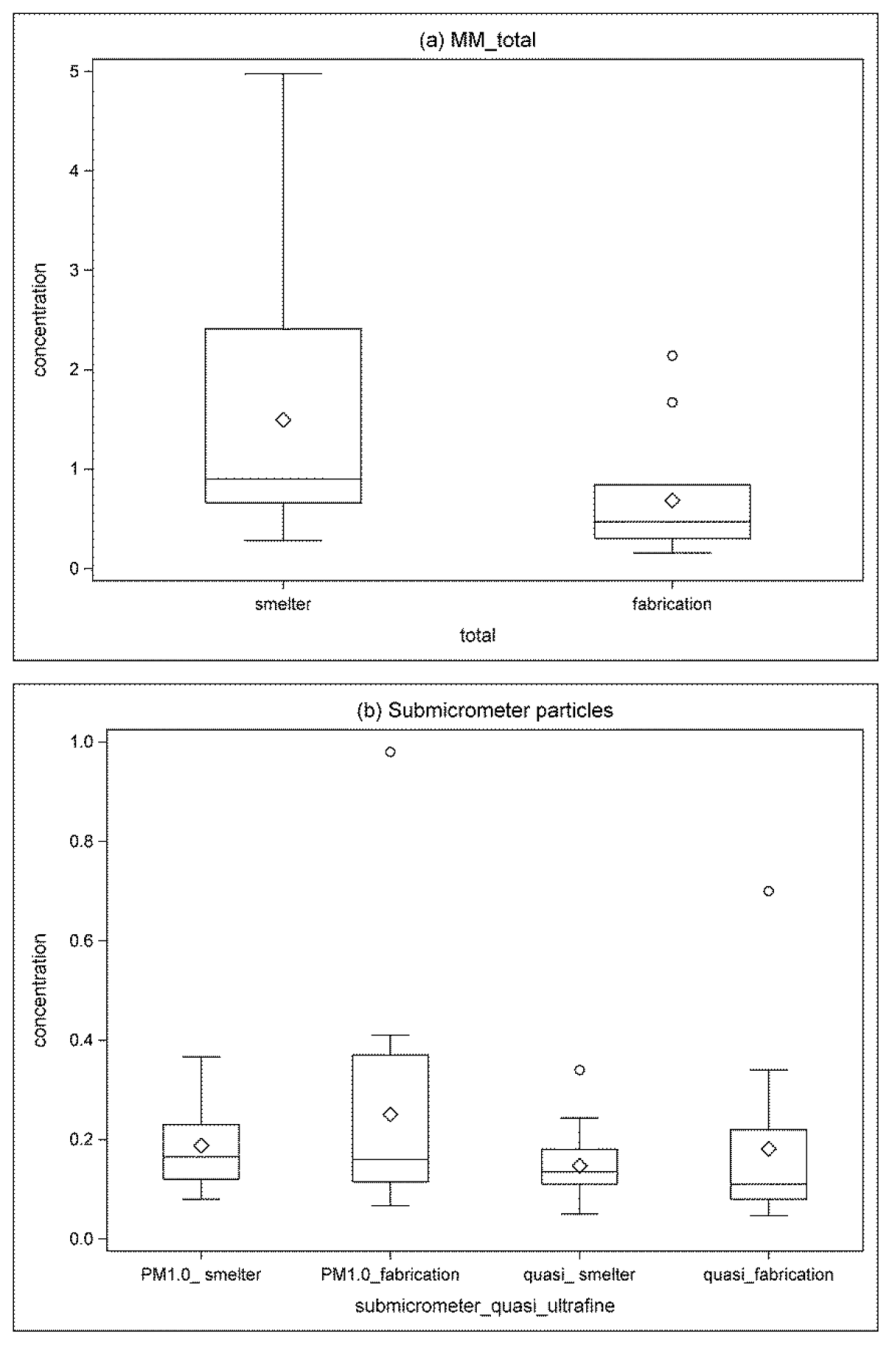
Box and whiskers plots showing particle concentrations (mg/m^3^) by particle size and facility type (smelter vs. fabrication), as measured by MiniMOUDI. (a) total particles (MM_total); (b) Submicrometer particles (PM_1.0_ and quasi-ultrafine particles). Boxes extend from the 25th to the 75th percentile, horizontal bars inside the boxes represent the median, diamonds inside the boxes represent the mean, whiskers extend to maximum and minimum observations within 1.5 times the length of the intra-quartile range (IQR) above and below the 75th and 25th percentiles, respectively, and outliers are represented as circles.

In contrast, both particle size and concentration were much less variable in the fabrication units than in the smelters. Particle size was significantly smaller in fabrication units than smelters (p = 0.001), with AM of MMAD equal to 1.31 (range: 0.39 – 2.27 μm). MMADs within the same department were similar at different locations or for different tasks. MiniMOUDI_total did not vary substantially in fabrication units, almost always less than 1.0 mg/m^3^ except at two locations (Table 1). Although total particle concentration was significantly higher in smelters than in the fabrication units (AM = 1.50 mg/m^3^ vs. 0.69 mg/m^3^, p < 0.05), the difference in concentrations diminished in sub-micrometer and quasi-ultrafine particle size ranges (Figure 3) because particles in fabrication units were predominantly small and the fraction of PM_10_ which was PM_1.0_ or PM_0.56_ was significantly higher in the fabrication units than in smelters (p < 0.001) (Table 1).

## 4. Discussion

Our data indicated that the reproducibility of PMI and MiniMOUDI cascade impactors ranged from moderate to high. The precision for PM_10_ was slightly better than for PM_3.2_ (MiniMOUDI) or PM_2.5_ (PMI). The comparison between PMI and MiniMOUDI revealed that the precision for PM_2.5_ (PMI) was very similar to that for PM_3.2_ (MiniMOUDI). As to the validity, PMI_PM_2.5_ was highly correlated with MM_PM_2.5_ (r = 0.97) and the slope of the regression line was 1.03. The agreement between PMI_PM_2.5_ (measured) and MM_PM_2.5_ (calculated) became not as strong as before, when two high PM_2.5_ concentration data points were not included, but they were still correlated (r=0.71). We do not consider those two measurements as outliers in our sampling data. The comparison between these two types of impactors may need more investigation for work settings with low PM concentrations or different particle size distributions. Total particle concentrations measured by PMI, MiniMOUDI and cassette did not agree well (not presented), which was not surprising, due to the effect of entry efficiency of these instruments. We did not observe the difference between PMI samples with first stage oiled versus not oiled, probably due to the property of the particles. Only limited numbers of oiled/non-oiled sample pairs were collected and most of them were collected at the fabrication processes where oil mist was the primary exposure. Both MiniMOUDI and PMI have been used to measure size selective particle air concentrations in environmental and occupational air pollution studies (Haynes et al., 2012; Hicks et al., 2012). However, neither the precision nor the validity of these impactors has been previously reported in published literature. Our findings contribute to the industrial hygiene knowledge of these instruments and can be used to help interpret similar measurement data in future studies.

We found that particle size distribution varied substantially in the production areas where area samples were collected. During aluminum manufacturing, workers are exposed to particles at each step from bauxite mining and refinery to smelting and metal materials fabrication. The central process of the aluminum smelting is electrolytic reduction, during which raw materials such as alumina, cryolite (Na3AlF6) and other salts are heated at high temperature (980°C) in electrolytic cells (pots). Other two important processes in the aluminum smelters are anode and cathode making that involve mixing, heating and baking of anthracite, graphite, calcined petroleum coke, coal-tar pitch and other materials. The processes release air pollutants such as carbon monoxide, carbon dioxide, particulate and gaseous fluorides, sulfur dioxide, polycyclic aromatic hydrocarbons and particulates. Whereas fabrication involves using alloying, casting, rolling, extruding, forging, drawing and traditional machining operations to make aluminum alloys, tubes, plates, sheets, foil and other final specialty products. The primary exposures in fabrication are oil mist and metals. Therefore, total particulate concentrations have been traditionally observed to be higher in smelters than in fabrication facilities, which is consistent with what we observed in our personal samples (Noth et al., 2014) as well as area samples (Table 1).

However, depending on the processes from which particles are generated, the particle size may vary differently from the concentrations. In our study, we found that particle size distributions were distinctively different at the processes in the smelting versus the fabricating facilities. Particles were significantly larger in most areas sampled in the smelters than those in the fabrication units. Although particle air concentrations have been observed to be significantly higher in smelters than in fabrication units when total particle or PM_2.5_ were measured (Noth et al., 2014), MiniMOUDI data indicate that the fraction of PM_10_ (MiniMOUDI) that was PM_1.0_ or PM_0.56_ was significantly higher in the fabrication units than those in smelters and thus the sub-micrometer and quasi-ultrafine particle concentrations were actually similar in smelting and fabricating facilities. In a companion report, ischemic heart disease incidence was associated with recent PM_2.5_ exposure; the hazard ratio rose to 1.5 in both smelter and fabrication facilities, though the analysis yielded stronger evidence of exposure-response in fabrication compared to smelting facilities, personal exposure was almost an order of magnitude higher in smelters (Costello et al., 2014). Ultrafine particle exposure in smelters in the aluminum industry has been of concern as processes in the potrooms have been reported to generate substantial amounts of particles with elevated ultrafine particle number concentrations (Thomassen et al., 2006; Debia et al., 2012). However, processes in fabrication units can be potential sources of ultrafine particles as well. For example, ultrafine particles can be generated when metal working fluids are applied on hot metal surfaces during rolling and extrusion. To date, investigation of size specific particle exposure in the aluminum industry has focused on potrooms in smelters (Hoflich et al., 2005; Thomassen et al., 2006; Weinbruch et al., 2010; Debia et al., 2012; Skaugset et al., 2014). No particle size selective exposure data have been reported for the fabrication facilities. The findings of the current study suggest that fabrication facilities may have the same level of ultrafine particle exposure as smelters, despite the fact that particle concentrations in fabrication units have traditionally been considered to be lower based on measured total particle concentrations.

## 5. Conclusions

We examined the reproducibility of two types of cascade impactors, MiniMOUDI and PMI, based on co-located area samples collected in eight diverse aluminum production facilities. We found the precision for the impactors ranged from moderate to high, with most of CVs less than 30%. Moreover, fine particles measured by PMI were found to be valid estimates of fine particle concentrations measured by MiniMOUDI. Considerable variations in particle size distribution and particle concentration were observed among production processes. Particle size and concentration varied substantially among departments in smelters, e.g., in the anode department from green anode to baked anode, but were similar in potrooms across facilities. In contrast, particle size and concentrations were much less variable in fabrication units. Particle size was significantly smaller in fabrications than in most areas of smelters. Although the area concentration of total particles was significantly higher in smelters than in fabrications, the area concentrations of sub-micrometer and quasi-ultrafine particles were similar, indicating exposure to ultrafine particles may be similar in smelters and fabrications.

## Figures and Tables

**Table 1:** Particle size distribution at different production areas (MiniMOUDI data)

Facility ID	Facility type^a^	Department	Task/Area	n	MMAD, um	GSD	MM total		MM_PM_10_		MM PM_1.0_		MM PM_0.56_		PM_1.0_/PM_10_		PM_0.56_/PM_10_	
					-		Conc^b^	CV^c^	Conc^b^	CV^c^	Conc^b^	CV^c^	Conc^b^	CV^c^	Ratio	CV^c^	Ratio	CV^c^
A	R	EHS	Welding	3	1.3	4.1	0.58	8%	0.44	15%	0.25	16%	0.17	15%	0.58	1%	0.38	11%
A	R	Raw Materials	General/“A” Tower Interchange	2	5.3	2.7	8.17	38%	2.05	40%	0.37	27%	0.09	67%	0.18	13%	0.05	100%

B	S	Utility Services	Bath crusher tending	3	2.5	2.8	1.88	17%	1.51	23%	0.23	30%	0.17	31%	0.15	19%	0.11	20%
B	S	Potroom	General	3	2.3	3.3	1.81	15%	1.23	15%	0.37	22%	0.24	25%	0.30	8%	0.20	11%
B	S	Potroom	General	3	2.3	3.3	0.70	16%	0.49	21%	0.15	35%	0.11	41%	0.30	18%	0.22	27%
B	S	Potroom	General	4	2	3.4	0.67	25%	0.52	23%	0.15	16%	0.13	14%	0.30	16%	0.25	22%
B	S	Baked Anode	General	3	3.1	3.2	2.77	39%	1.60	26%	0.37	8%	0.34	9%	0.24	26%	0.22	27%
C	S	Aluminum Services	General/Bath crusher	2	5.6	2.6	4.98	31%	3.10	23%	0.27	147%	0.24	166%	0.08	136%	0.07	158%
C	S	Potroom	General	2	2.6	2.7	0.78	14%	0.65	8%	0.08	0%	0.05	40%	0.12	8%	0.08	33%
C	S	Potroom	General/Potroom10, Line5	2	1.9	3.5	0.97	23%	0.75	26%	0.29	32%	0.17	35%	0.38	6%	0.23	10%
C	S	Potroom	General	2	1.7	3.1	1.29	5%	1.05	9%	0.31	16%	0.25	32%	0.29	25%	0.24	40%
C	S	Green Anode	General/Greenmill Running	1	5.6	2.7	2.71	-	0.67	-	0.11	-	0.05	-	0.16	-	0.07	-
C	S	Green Anode	General	1	4.2	2.6	3.03	-	1.63	-	0.12	-	0.06	-	0.07	-	0.04	-
C	S	Green Anode	General	1	4.2	2.6	2.41	-	1.23	-	0.08	-	0.06	-	0.07	-	0.05	-
C	S	Baked Anode	Bricksaw operation	3	1	4.3	0.28	9%	0.23	4%	0.12	8%	0.11	9%	0.52	7%	0.48	8%
C	S	Baked Anode	General	1	0.9	5.3	0.38	-	0.27	-	0.19	-	0.18	-	0.70	-	0.67	-
C	S	Baked Anode	General	1	0.7	4.4	0.42	-	0.37	-	0.19	-	0.18	-	0.51	-	0.49	-

C	F	Hot Mill	General/Continuous Mill	1	0.9	4.4	0.84	-	0.73	-	0.41	-	0.34	-	0.56	-	0.47	-
C	F	Hot Mill	General/Reversing Mill	1	0.8	4.5	1.67	-	1.54	-	0.98	-	0.70	-	0.64	-	0.45	-
D	F	Hot Rolling	Fork Truck Operator/220” Pulpit	1	1.2	4.2	0.84	-	0.62	-	0.37	-	0.24	-	0.60	-	0.39	-
D	F	Hot Rolling	Shear Operation/8”Shear	1	1.1	4.1	0.36	-	0.31	-	0.16	-	0.13	-	0.52	-	0.42	-
D	F	Hot Rolling	Shear Operation/3”Shear	1	1.6	3.9	0.47	-	0.33	-	0.17	-	0.10	-	0.52	-	0.30	-
D	F	Cold Rolling	Cold Mill Operation/816/#1 cold Mill Exit	1	1.4	3.4	0.30	-	0.27	-	0.10	-	0.08	-	0.37	-	0.30	-
E	F	Inspection	General	2	1.5	3.9	0.40	10%	0.30	0%	0.14	7%	0.11	10%	0.45	7%	0.35	10%
F	F	Plate Mill	Sawing/902W/#2 Alu-Cut Saw	2	1.8	3.4	0.64	43%	0.48	33%	0.15	21%	0.13	24%	0.31	13%	0.26	10%
F	F	Ingot Plant	Ingot casting/810/4 DC Pit	1	2.3	3.1	2.14	-	1.58	-	0.37	-	0.22	-	0.23	-	0.14	-
G	F	101 Metal Cells	General/EQUIAX	3	0.8	4.6	0.17	3%	0.15	18%	0.09	38%	0.08	38%	0.59	20%	0.50	19%
H	F	Gate Removal	General/PLANT 10	1	1	3.8	0.41	-	0.35	-	0.17	-	0.12	-	0.49	-	0.34	-
H	F	Gate Removal	General/PLANT 10	1	1.7	3.2	.	-	0.16	-	0.06	-	0.02	-	0.38	-	0.13	-
H	F	Gate Removal	General/PLANT 1	1	1.5	3.3	0.21	-	0.18	-	0.06	-	0.04	-	0.33	-	0.22	-
H	F	Gate Removal	General/PLANT 1	2	1.3	3.7	0.13	15%	0.12	0%	0.07	86%	0.05	120%	0.58	86%	0.42	120%

a Facility type, R = refinery; S = smelter; F = fabrication.

b Particle air concentration, in mg/m^3^.

c Coefficient variation of collocated samples (relative difference for duplicate samples and “-” for single sample at a location).
